# Quantifying the Gap between Expected and Actual Rates of Antibiotic Prescribing in British Columbia, Canada

**DOI:** 10.3390/antibiotics10111428

**Published:** 2021-11-22

**Authors:** Ariana Saatchi, Ji-Won Yoo, Kevin L. Schwartz, Michael Silverman, Andrew M. Morris, David M. Patrick, James McCormack, Fawziah Marra

**Affiliations:** 1 Faculty of Pharmaceutical Sciences, University of British Columbia, Vancouver, BC V6T 1Z3, Canada; ariana.saatchi@ubc.ca (A.S.); jwdanielyoo@gmail.com (J.-W.Y.); james.mccormack@ubc.ca (J.M.); 2 Public Health Ontario, Toronto, ON M5G 1V2, Canada; kevin.schwartz@oahpp.ca; 3 Dalla Lana School of Public Health, University of Toronto, Toronto, ON M5T 3M7, Canada; 4 Lawson Health Research Institute, London, ON N6A 4V2, Canada; michael.silverman@sjhc.london.on.ca; 5 Faculty of Medicine, University of Western Ontario, London, ON N6A 5C1, Canada; 6 Sinai Health System, University Health Network and University of Toronto, Toronto, ON M5G 1L7, Canada; andrew.morris@sinaihealth.ca; 7 British Columbia Centre for Disease Control, Vancouver, BC V5Z 4R4, Canada; david.patrick@bccdc.ca; 8 School of Population and Public Health, University of British Columbia, Vancouver, BC V6T 1Z3, Canada

**Keywords:** antibiotics, epidemiology, antimicrobial resistance (AMR), prescription, respiratory tract infections, outpatient care, emergency care, British Columbia, Canada

## Abstract

Despite decades of stewardship efforts to combat antimicrobial resistance and quantify changes in use, the quality of antibiotic use in British Columbia (BC) remains unknown. As the overuse and misuse of antibiotics drives antibiotic resistance, it is imperative to expand surveillance efforts to examine the quality of antibiotic prescriptions. In late 2019, Canadian expected rates of antibiotic prescribing were developed for common infections. These rates were utilized to quantify the gap between the observed rates of prescribing and Canadian expected rates for antibiotic use for the province of BC. The prescribing data were extracted and matched to physician billing systems using anonymized patient identifiers from 1 January 2000 to 31 December 2018. Outpatient prescribing was further subdivided into community and emergency department settings and stratified by the following age groups: <2 years, 2–18 years, and ≥19 years. The proportions of physician visits that received antibiotic prescription were compared against the Canadian expected rates to quantify the unnecessary use for 18 common indications. Respiratory tract infections (RTI), including acute bronchitis, acute sinusitis, and acute pharyngitis, reported significant levels of overprescribing. Across all ages and health care settings, prescribing for RTI indications occurred at rates 2–8 times higher than the expected rates recommended by a group of expert Canadian physicians. Understanding the magnitude of unnecessary prescribing is a first step in delineating the provincial prescribing quality. The quantification of antibiotic overuse offers concrete targets for provincial stewardship efforts to reduce unnecessary prescribing by an average of 30% across both outpatient and emergency care settings.

## 1. Introduction

The misuse of antibiotics is a global crisis. Over 30% of the antibiotic prescriptions in the United States were deemed inappropriate, and one third of the antibiotic prescriptions for upper respiratory tract infections (URTI) in Europe had no justification for their use [1,2]. There is an urgent need for antimicrobial stewardship: a study in the United Kingdom (UK) suggested a 50% reduction in inappropriate prescribing is required to quell the increasing rates of AMR [3]. In the UK, prescribing guidelines coupled with expert opinion have been used to quantify the gap between the current prescribing practices and the expected antibiotic use [4,5]. These rates offer a concrete benchmark against which to weigh indication-specific antibiotic use and characterize the quality of prescribing. The expected rates for UK primary care have been available since 2018 and have been utilized to quantify inappropriate and unnecessary antibiotic use across many common indications, including urinary tract as well as upper and lower respiratory tract infections [5].

Antibiotic use is ubiquitous globally, as well as within Canada, and, while effective for specific indications, the overuse and misuse of antibiotics has been strongly associated with the increasing rates of antimicrobial resistance (AMR) [6,7]. Antibiotic use in the outpatient or emergency setting is often contingent on patient factors not captured within administrative health data [8]. The establishment of expected, or maximal, prescribing rates offers a benchmark against which prescriptions issued may be contextualized. Although limitations, such as participant self-selection, physician biases to limited patient populations, and issues with reliability, sensitivity, and/or specificity, are inherent in their generation, benchmark rates offer valuable insight regarding the ongoing blackbox of prescribing quality [9,10]. In this regard, point prevalence surveys may be useful in delineating further data on the appropriateness of antimicrobial prescribing [11,12,13]. In late 2019, Canadian rates of expected antibiotic prescribing were developed for common infections in the outpatient setting [14]. We performed this retrospective cohort study to quantify the gap between the observed rates of prescribing and the Canadian expected rates for antibiotic use for the province of BC. Our objective was to uncover indication-specific rates of potential unnecessary prescribing as the data may direct specific stewardship efforts to reduce suboptimal antibiotic use and inhibit the development of AMR.

## 2. Results

Over the 19-year study period, we had a total of 3,490,585 unique patients, with an average of 447,107 unique patients per year prescribed an antibiotic for one of the 18 included indications across both community and emergency care (Table 1).

The data for 78,495,265 physician encounters were extracted between 1 January 2000 and 31 December 2018. Outpatient care was responsible for 77,754,597 of the cases over the 19-year study period, with 7,708,016 unique patients. Emergency care accounted for an additional 740,668 physician encounters between 2012 to 2018, attributed to 289,954 unique patients. Across both healthcare settings, 57% of all antibiotic dispensation records linked to an indication within the study scope. Antibiotics were more often prescribed for females in urban settings, particularly in the Fraser health authority, and a negative association was observed between increased antibiotic use and income quintile.

**Figure 1:**
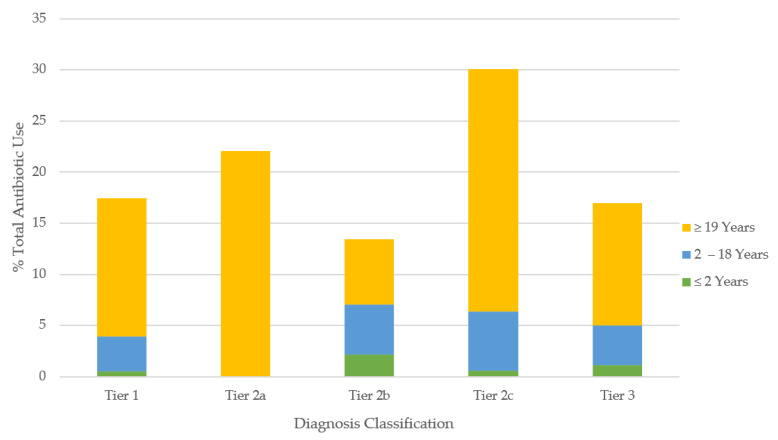
Proportion of all antibiotics prescribed for common infections according to tier classification. Tier 1 = antibiotics always indicated (maximal rate 100%); tier 2a = antibiotics frequently indicated (maximal rate: 51–99%); tier 2b = antibiotics sometimes indicated (maximal rate: 21–50%); tier 2c = antibiotics rarely indicated (maximal rate: 1–20%); tier 3 = antibiotics never indicated (maximal rate: 0%).

The most common diagnoses in outpatient care were the common cold, UTI, asthma, and purulent SSTI, making up 53% of the cases (Table 2). In contrast, purulent and non-purulent SSTI, UTI, and pneumonia made up 59% of the emergency cases. The overall antibiotic use was most elevated for the indications classified within tier 2c, followed by tier 2a, tier 1, and tier 3, with the least prescriptions issued for tier 2b diagnoses (Table 2; Figure 1).

### Unnecessary Antibiotic Utilization

Table 2 shows that roughly 30 million antibiotics were prescribed over the 19-year study period, with 28,682,721 antibiotics prescribed in outpatient care and 419,602 antibiotics prescribed in emergency care. The overall antibiotic prescribing rate for all 18 indications was 37% in outpatient care, and 66% in emergency settings. The diagnoses involved with the most outpatient antibiotic prescriptions were UTI (23%), the common cold (13%), and bronchitis (12%). In emergency care, non-purulent SSTI (26%), UTI (22%), and pneumonia (14%) led the diagnoses.

The potential unnecessary antibiotic prescribing is shown in Table 3. In outpatient care, bronchitis (52%), dental conditions (50%), acute sinusitis (48%), and acute pharyngitis (42%) had the highest rates of unnecessary antibiotic prescribing. Similarly, emergency care reported overprescribing for dental conditions (75%), acute sinusitis (52%), and bronchitis (49%).

**Figure 2:**
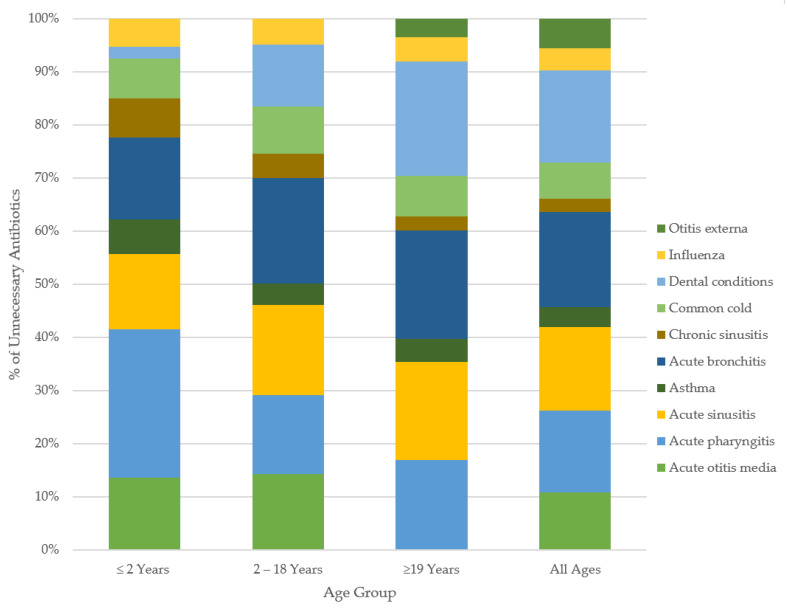
Overall percent unnecessary antibiotic use for common conditions, by age. Only those tier2a,2b,2c, 3 conditions prescribed at rates above the Canadian maximal were included in our classification of unnecessary prescribing. Tier 1 indications were restricted within analyses to an unnecessary prescribing rate of 0%.

**Figure 3:**
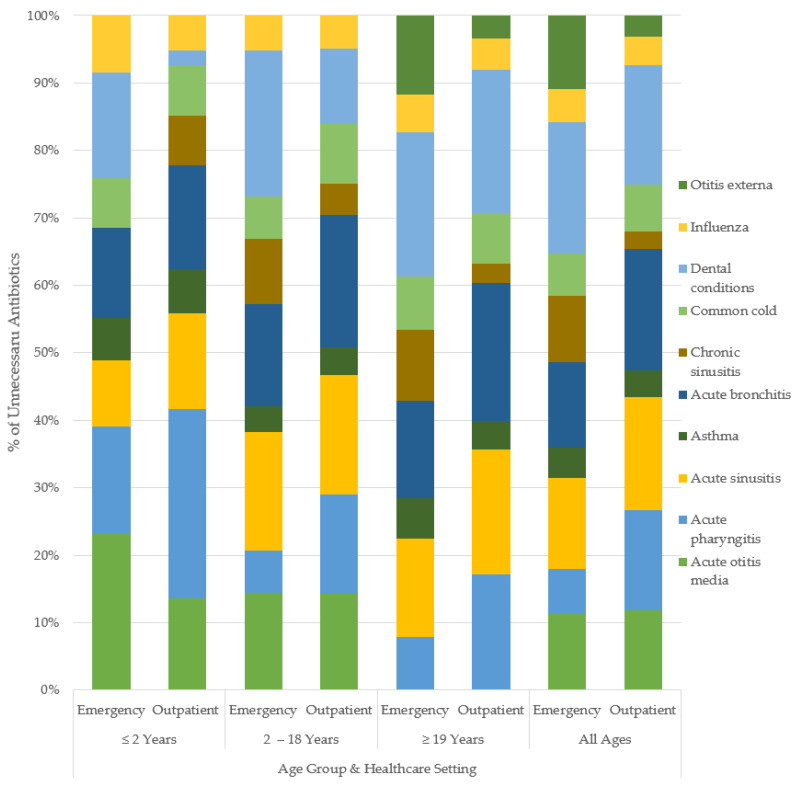
Percent unnecessary antibiotic use for common conditions, by age and healthcare setting.

By age groups in outpatient care: patients <2 years of age were the most overprescribed for acute pharyngitis (59%), while bronchitis led overprescribing for those aged 2–18 years, with 49.8% unnecessary. For adult patients (≥19 years), dental infections (56%) received the highest rate of unnecessary antibiotic prescription. The trends by age in emergency care varied from the community prescribing patterns. The patients aged <2 years were overprescribed for AOM at the highest rate (45%), while dental infections (65%) were overprescribed the most in those aged 2–18 years. The highest rate of unnecessary antibiotic use in adults was 76%, for dental conditions. The magnitudes of unnecessary antibiotic use varied across healthcare settings with similar directional trends across indications. Figure 2 and Figure 3 show the breakdown of the percent of unnecessary prescribing for each indication by age category and healthcare setting.

## 3. Discussion

Elevated, unnecessary antibiotic use was most pronounced in respiratory tract indications (RTI) overall, including: acute bronchitis (53%), acute sinusitis (46%), and acute pharyngitis (46%), wherein all three age categories were prescribed in excess of at least 30%. Of these RTI conditions, children aged less than 2 years received the most unnecessary prescriptions for acute pharyngitis, while adult patients led bronchitis and sinusitis. Moreover, as respiratory tract infections accounted for over 60% of all the physician visits, and 55% of all the associated antibiotic use, the scale of unnecessary use for these indications translates to high levels of inappropriate prescription in both the community and emergency healthcare settings. Across all the indications and both healthcare settings, prescribing for adults was 29% unnecessary, with 28% unnecessary for children aged 2–18 years and 24% unnecessary for children aged less than 2 years.

Our study corroborates findings from several studies across jurisdictions operating under similar universal healthcare systems, such as the United Kingdom, and other provinces within Canada [15,16]. When compared against the expected prescribing rates, Pouwels et al. found that the outpatient antibiotic use in the UK was highly elevated for RTI [5]. This elevated trend is shared across two Canadian provinces: British Columbia and Ontario had comparable rates of overprescribing identified across both Canadian healthcare systems for RTI. In Ontario outpatient care, Schwartz et al. report a 53% rate of overprescribing for bronchitis, while, in BC, antibiotics were overprescribed by 52% and 49% in the outpatient and emergency care settings, respectively, for the same indication [16]. One possible explanation underlying these suboptimal prescriptions could be perceived patient expectations by physicians to prescribe in the outpatient setting despite self-limiting or non-bacterial etiologies [17]. Although clinical guidelines do not recommend the use of antibiotics for tier 2c (antibiotics rarely indicated) and/or tier 3 (antibiotics never indicated) URTI, physicians may still prescribe based on the symptoms and severity of clinical presentation [17].

Although dental conditions account for a minority of the total prescribing (1%), the elevated proportion of unnecessary prescribing in adults is concerning. BC emergency care had a 75% rate of overprescribing for dental conditions, far more than the 32% seen in ON outpatient care or the 50% in BC outpatient care. However, as dental conditions are a tier 2c indication, with a 4% expected rate of prescription across all ages, unnecessary antibiotic use is significant for this diagnosis regardless of the healthcare setting. While most medical care is publicly funded in BC and essentially universal in Canada, dental services remain a privately insured sector of healthcare. As such, dentists in BC do not bill MSP directly, nor were they explicitly identified within NACRS (emergency) analyses. The overprescribing reported is not related to dental practitioners but rather physicians treating dental conditions—as further indicated by the reduced number of overall physician visits for dental conditions in comparison to other indications. However, inappropriate antibiotic use in the treatment of dental conditions, in both community and emergency settings, has been well documented across BC, the United States, the United Kingdom, as well as Australia [18,19,20]. Patients with inadequate dental insurance coverage might disproportionately seek care from physicians to treat dental conditions, resulting in the overuse of antibiotics offsetting under-pursued surgical interventions. Although the elevated use of antibiotics in dentistry has been documented in the literature, the results reported here cannot be extrapolated to dental practice.

The expected Canadian rates of prescribing were engaged within this study as maximal rates throughout analyses—wherein only instances of excess prescribing were characterized as unnecessary. The decision to disregard the rates of prescribing below expected was founded in the principles of antimicrobial stewardship [21,22]. In the absence of reported patient harms, it would be misguided to interpret any gap between the expected and actual rates of antibiotic prescription as an issue of under-prescribing. Moreover, if lower rates of antibiotic use have not resulted in increased negative patient outcomes, then the current prescribing norms underlying the Canadian expected rates can be called into question. On that point, Gulliford et al. report no association between lower rates of antibiotic use and patient safety outcomes [23]. Of the 18 indications reviewed in this study, only 10 conditions were discussed with respect to unnecessary prescribing, with the majority of urinary tract and skin/soft tissue diagnoses ruled out of subsequent analyses as negative magnitudes of difference between the expected and observed prescribing were restricted to 0% unnecessary. With that said, the conclusions drawn are twofold: first, further research to confirm the incidence of adverse outcomes in the event of lower rates of antibiotic use is needed in BC, and, secondly, the expected rate generation dependent on expert opinion may be inherently elevated as a result of the current practice norms on which they are based.

Our limitations are similar to other retrospective studies conducted using administrative data. The linkage of PharmaNet dispensing records and physician billing data may exclude refill prescriptions as the patient would not visit the clinic. Further, our rates do not account for unfilled prescriptions issued, and the levels of compliance to the dispensed medications are unknown. Throughout the study period, 57% of all the antibiotic use was linked to an indication within the study scope, with an additional 22,193,615 antibiotic dispensations unlinked and excluded from further analyses. These prescriptions can be attributed to indications beyond the study scope, hospital discharge records, prescription refills, and/or non-physician healthcare providers (e.g., dentists, naturopaths). Moreover, the records of indications are reliant on accurate coding by billing physicians; in the absence of lab data to confirm the bacterial etiology, our use of ICD-9/10 codes may be subject to misclassification bias. Although it is notable that Canadian primary-care physician claims data have a high positive-predictive value for the diagnosis of some common infections, including acute non-bacterial upper-respiratory infections, concerns regarding the specificity of ICD-9/10 codes remain [24,25]. Furthermore, characterizations of unnecessary antibiotic use do not account for patient comorbidities, concurrent medications, allergies, or other relevant factors that might justify otherwise inappropriate antibiotic use; however, these patient factors do not explain the inter-physician variability in antibiotic prescribing [26]. The scope of the data available through the NACRS is also quite marginal in tandem with the reduced temporal period. The BC Ministry of Health mandated the introduction of NACRS data reporting in 2010, with 15 high-volume emergency departments (ED) included at inception. By 2013, 29 EDs in BC reported to NACRS; however, the index remains new within the province, and the rates reported are likely an under-estimate of the true ED rates of prescribing. Moreover, in comparing rates of unnecessary antibiotic use across healthcare settings, the NACRS and MSP do not encompass the same time periods as emergency department data in the NACRS started collection recently. This difference is notable as direct comparisons of prescribing across healthcare settings do not account for stewardship interventions, guideline, and/or formulary changes over time.

Research has identified a strong association between antibiotic overprescribing and subsequent antimicrobial resistance [27,28,29,30,31]. Recent data from the World Health Organization and the Centers for Disease Control show continued increasing resistance with Gram-positive pathogens, such as Streptococcus pneumoniae and Staphylococcus aureus. In addition, the resistance in Gram-negative pathogens, such as *E. coli*, *Klebsiella* spp., and other Enterobacteriae, continues to rise due to the acquisition of extended spectrum beta-lactamases (ESBL) and carbapenenases; interventions to protect the efficacy of these essential medications is paramount [32,33,34]. Moreover, antibiotics can lead to acute adverse patient events and outcomes, including drug allergies, and can lead to long-term complications associated with perturbed microbiota [35]. With these risks in mind, and to protect the efficacy of these essential medications, the DBND program in BC aims to reduce unnecessary prescribing and optimize antibiotic use [36,37]. High caliber stewardship efforts aim to ensure that antibiotics are available when medically necessary and reserved otherwise to protect against rising resistance. A recent study confirmed that the overall antibiotic prescribing rates are declining in BC [38]. As further reductions remain a target for provincial stewardship, this study offers the first concrete quantifications for the potential reduction in unnecessary antibiotic use within BC for 18 distinct indications across two healthcare settings.

## 4. Materials and Methods

### 4.1. Data Sources

The Ministry of Health in British Columbia houses several healthcare-related databases, which have comprehensive information on BC residents (population: 5 million) [39]. Antibiotic information was extracted from BC PharmaNet, a centralized data system that links all pharmacies with every prescription dispensed through community and hospital outpatient pharmacies [40]. All antimicrobials are recorded in this system except those used for treatment of sexually transmitted infections and HIV, as well as medications administered within the hospitals/emergency departments. The Medical Service Plan (MSP) billing system records all claims submitted by physicians for services provided to BC residents, including diagnostic codes [41]. The National Ambulatory Care Reporting System (NACRS) captures data from 29 high volume emergency departments across BC [42]. Established in 2012, NACRS data are included from index inception onwards. Patient demographics were supplied through a consolidation file containing age and sex information [43]. Data were extracted, anonymized, and made available to researchers by Population Data BC. All inferences, opinions, and conclusions drawn in this study are those of the authors and do not reflect the opinions or policies of the data steward(s).

### 4.2. Study Population

Our study included all BC residents from 1 January 2000 to 31 December 2018. Physician visit and indication data were pulled from both MSP (outpatient care) and NACRS (emergency care) indices, and then matched to antibiotic dispensations extracted from PharmaNet using anonymized patient identifiers. A prescription and diagnosis were linked using an algorithm that matched the date on which the medication was dispensed to a practitioner service date within 5 days prior. If a practitioner service date was associated with more than one diagnostic code, or multiple service dates fell within a single 5-day period of a prescription dispensing date, then a three-tiered hierarchy was applied to link only the most relevant diagnostic code to the prescription. If multiple diagnoses were listed from the same tier, the first physician code was selected for analysis. Multiple prescriptions per subject were permitted in our analyses. Antibiotic data that did not match to a physician visit record for an indication within study scope were not included in the analyses. All data outputs with *n* < 5 were excluded from subsequent analyses to preserve subject anonymity.

### 4.3. Canadian Expected Prescribing Rates

A study by Wu et al., published in 2020, presented Canadian expected rates of outpatient prescribing for the incidence of various common infections [14]. These rates were generated through expert opinion elicitation. In the absence of thorough clinical guidelines, the modified Delphi method has increasingly become a common tool in medical research to assist in delineating appropriate, or expected, markers for diagnosis and/or treatment [44]. The expected rates discussed within this paper are essentially the maximal (i.e., upper-limit) prescribing rates, stratified by three age groups (<2 years, 2–18 years, >19) for common clinical conditions.

We have mirrored their methodology to generate comparable rates of observed prescribing in BC. Furthermore, data were extracted for 18 of the same common clinical conditions they reviewed: acute otitis media (AOM), acute pharyngitis, acute sinusitis, asthma, bronchitis, chronic sinusitis, common cold, dental conditions, epididymo-orchitis, influenza, non-purulent soft tissue skin infections (SSTI), otitis externa, pneumonia, prostatitis, purulent SSTI, pyelonephritis, reproductive tract infections, and urinary tract infections (UTI); the exception was chronic obstructive pulmonary disease (COPD) as their expected prescribing rate is for acute exacerbations of COPD, which is not well-captured by billing codes. These indications were further organized into 5 tiers based on the proportion of expected prescribing. Tier 1 included diagnoses for which antibiotics are routinely used (i.e., expected prescribing rate of 100%). Tier 2 were diagnoses for which antibiotics are frequently indicated (tier 2a: 51–99% expected prescribing rate), sometimes indicated (tier 2b: 21–50% expected prescribing rate), or rarely indicated (tier 2c: 1–20% expected prescribing rate). Finally, tier 3 included diagnoses for which antibiotic use is never indicated (0% expected prescribing rate).

### 4.4. Outcomes and Statistical Analyses

Antibiotics were classified based on the Anatomical Therapeutic Chemical (ATC) classification system developed by WHO [45]. Consumption rates were calculated as prescriptions per 1000 population per year, using age- and gender-specific denominator estimates for the population from Statistics BC [39]. MSP diagnostic codes are ordered by the ninth revision of the International Classification of Diseases developed by WHO, commonly referred to as ICD-9, while NACRS reports ICD-10 billing codes [46,47,48].

Overall rates of total antibiotic use were examined and then stratified by age group (<2, 2–18, ≥19 years). Observed rates were compared against maximal references, extracted from Canadian expected rates, and the difference in prescribing rates were calculated for each indication across both outpatient and emergency department care. Unnecessary antibiotic use was identified using the magnitude of difference between expected and observed prescribing by indication, age, and healthcare setting. Prescribing below expected rates was not classified as inappropriate, and all negative magnitudes of difference were subsequently reported to be: 0% prescribing.

## 5. Conclusions

This study reports elevated levels of unnecessary antibiotic use for RTI indications across all ages and two distinct healthcare settings. These diagnoses, commonly of viral etiology, do not often warrant the use of antibiotic treatment (tier 2c and/or 3), yet antibiotics continue to be prescribed at rates far above the expert and guideline recommendations when compared to the expected rates. The magnitudes of the reported unnecessary prescribing offer new, actionable targets for provincial stewardship efforts.

## Figures and Tables

**Table 1:** Cohort characteristics.

	Outpatient Care Setting 2000–2018	Emergency Care Setting 2012–2018
Total number of patients	3,240,894	249,691
Average patients per year	405,685	41,422
Age (years)		
Mean	41.62	45.17
Standard Deviation	25.44	24.95
<2 Years	378,927 (4.92%)	14,600 (5.04%)
2–18 Years	1,520,761 (19.73%)	40,057 (13.81%)
≥19 Years	5,808,328 (75.35%)	235,297 (81.15%)
Sex		
Female	4,792,943 (62.18%)	165,137 (56.95%)
Male	2,914,119 (37.81%)	124,807 (43.04%)
Income Quintile		
Quintile 1 (lowest)	1,644,735 (21.34%)	71,376 (24.62%)
Quintile 2	1,589,201 (20.62%)	61,103 (21.07%)
Quintile 3	1,516,110 (19.67%)	56,650 (19.54%)
Quintile 4	1,465,429 (19.01%)	52,771 (18.2%)
Quintile 5 (highest)	1,349,927 (17.51%)	43,419 (14.97%)
Missing	133,309 (1.73%)	3498 (1.21%)
Geographic Region		
Rural	1,274,318 (16.53%)	10,800 (3.72%)
Urban	6,139,403 (79.65%)	268,037 (92.44%)
Missing	294,295 (3.82%)	11,117 (3.83%)
Health Authority		
Interior	1,259,910 (16.35%)	4879 (1.68%)
Fraser	2,968,609 (38.51%)	164,177 (56.62%)
Vancouver Coastal	1,648,260 (21.38%)	88,229 (30.43%)
Vancouver Island	1,314,339 (17.05%)	21,634 (7.46%)
Northern	493,305 (6.4%)	9912 (3.42%)
Missing	15,968 (0.21%)	768 (0.26%)

**Table 2:** Number of total cases and associated antibiotics dispensed per indication of interest, by healthcare setting.

Classification	Diagnosis	Outpatient Care Setting ^1^		Emergency Care Setting ^2^	
		Prescriptions Issued ^3^	Total Physician Visits ^4^	Prescriptions Issued	Total Physician Visits
Tier 1: *Always indicated (100%)*	Pneumonia	1,048,153 (3.65%)	2,525,744 (3.25%)	69,424 (14.12%)	97,538 (19.84%)
	Pyelonephritis	200,042 (0.70%)	395,291 (0.51%)	24,453 (4.97%)	31,131 (6.33%)
	Non-Purulent SSTI	2,668,652 (9.30%)	4,762,565 (6.13%)	132,539 (26.96%)	180,189 (36.65%)
	Reproductive tract	367,126 (1.28%)	2,502,766 (3.22%)	2576 (0.52%)	2754 (0.56%)
	Urinary tract infections (aged ≤18 y)	560,958 (1.96%)	957,393 (1.23%)	13,388 (2.72%)	16,034 (3.26%)
Tier 2a: *Frequently indicated* *(51–99%)*	Urinary tract infections (aged >18 y)	6,140,403 (21.41%)	9,724,013 (12.51%)	99,168 (20.17%)	118,149 (24.03%)
	Prostatitis	110,482 (0.39%)	381,996 (0.49%)	1713 (0.35%)	2312 (0.47%)
	Epididymo-orchitis	73,955 (0.26%)	173,088 (0.22%)	5268 (1.07%)	6947 (1.41%)
Tier 2b: *Sometimes indicated* *(21–50%)*	Purulent SSTI	1,443,819 (5.03%)	6,964,320 (8.96%)	33,650 (6.84%)	51,538 (10.48%)
	Acute Otitis Media	2,343,863 (8.17%)	3,899,643 (5.02%)	23,823 (4.85%)	31,265 (6.36%)
	Pharyngitis (aged ≤2 y)	81,486 (0.28%)	105,334 (0.14%)	619 (0.13%)	1267 (0.26%)
Tier 2c: *Rarely indicated* *(1–20%)*	Acute sinusitis	2,635,709 (9.19%)	3,990,765 (5.13%)	6281 (1.28%)	8949 (1.82%)
	Chronic sinusitis	404,106 (1.41%)	1,883,735 (2.42%)	535 (0.11%)	1068 (0.22%)
	Bronchitis	3,406,425 (11.88%)	5,746,837 (7.39%)	14,981 (3.05%)	26,263 (5.34%)
	Dental Conditions	353,089 (1.23%)	649,259 (0.84%)	19,911 (4.05%)	25,275 (5.14%)
	Otitis Externa (aged >18 y)	210,929 (0.74%)	2,121,058 (2.73%)	2874 (0.58%)	6707 (1.36%)
	Pharyngitis (aged >2 y)	1,696,442 (5.91%)	2,264,601 (2.91%)	20,718 (4.21%)	36,475 (7.42%)
Tier 3: *Never indicated* *(0%)*	Asthma	837,508 (2.92%)	7,493,929 (9.64%)	7875 (1.6%)	45,529 (9.26%)
	Common Cold	3,746,608 (13.06%)	18,788,085 (24.16%)	6685 (1.36%)	27,361 (5.57%)
	Influenza	218,802 (0.76%)	1,803,191 (2.32%)	4150 (0.84%)	21,578 (4.39%)
	Otitis Externa (aged ≤18 y)	134,164 (0.47%)	620,984 (0.80%)	971 (0.20%)	2339 (0.48%)
Overall	All Indications ^5^	28,682,721	77,754,597	491,602	740,668

^1^ Community care data extracted from Medical Services Plan database from 2000–2018; ^2^ ambulatory care data extracted from National Ambulatory Care Reporting System database from 2012–2018; ^3^ refers to the total number of physician visits linked to a record of antibiotic dispensation ±5 days; ^4^ refers to the total number of physician visits per indication; ^5^ refers to the sum total of 18 indications of interest within study scope.

**Table 3:** Rate of antibiotic prescribing between outpatient and emergency care, stratified by age.

Diagnoses	Age (yr); Number of Outpatient Care Visits				Age (yr); Number of Emergency Care Visits				Age (yr); Outpatient Prescribing rate/% *Unnecessary* ^1^				Age (yr); Emergency Prescribing rate/% *Unnecessary*			
	<2	2–18	≥19	All Ages	<2	2–18	≥19	All Ages	<2	2–18	≥19	All Ages	<2	2–18	≥19	All Ages
Acute otitis media	731,530	1,797,583	-	2,529,113	6546	16,367	-	31,265	68.8/*28.6*	66.0/*36.0*	-	60.1/*33.9*	84.6/*44.6*	73.4/*43.3*	-	76.2/*43.6*
Acute pharyngitis	105,334	1,047,467	1,217,134	2,369,935	1267	10,950	25,525	37,742	77.4/*59.4*	77.3/*37.3*	72.8/*44.8*	75.0/*42.2*	48.9/*30.9*	58.6/*18.6*	56.0/*28.0*	56.5/*25.4*
Acute sinusitis	57,725	435,409	3,497,631	3,990,765	86	584	8279	8949	49.7/*29.7*	62.7/*44.7*	66.7/*48.7*	66.0/*47.8*	38.9/*18.9*	72.8/*52.8*	70.5/*52.5*	70.3/*52.2*
Asthma	306,349	1,873,975	5,313,605	7,493,929	4148	14,629	26,752	45,529	14.0/*14.0*	10.2/*10.2*	11.3/*11.3*	11.2/*11.2*	12.0/*12.0*	11.3/*11.3*	21.4/*21.4*	17.3/*17.3*
Bronchitis	380,898	946,026	4,419,913	5,746,837	1665	2350	22,248	26,263	37.5/*32.5*	57.8/*49.8*	61.5/*53.5*	59.3/*51.5*	30.8/*25.8*	53.2/*45.2*	59.4/*51.4*	57.0/*49.2*
Chronic sinusitis	3055	73,952	1,806,728	1,883,735	-	42	1031	1073	29.5/*15.5*	25.4/*11.4*	21.3/*7.3*	21.5/*7.5*	-	42.9/*28.9*	51.9/*37.9*	51.5/*37.5*
Common cold	1,572,357	3,501,575	13,714,153	18,788,085	3550	5654	18,157	27,361	15.8/*15.8*	22.5/*22.5*	19.7/*19.7*	19.9/*19.9*	14.3/*14.3*	18.9/*18.9*	28.1/*28.1*	24.4/*24.4*
Dental Conditions	18,444	93,420	537,395	649,259	234	2473	22,568	25,275	8.5/*4.5*	32.3/*28.3*	59.8/*55.8*	58.4/*50.4*	34.2/*30.2*	68.5/*64.5*	80.4/*76.4*	78.8/*74.8*
Epididymo-orchitis	-	11,874	159,596	171,470	-	658	6274	6932	-	33.3/*0.0*	43.8/*0.0*	42.3/*0.0*	-	62.5/*0.0*	77.3/*0.0*	75.8/*0.0*
Influenza	94,316	382,918	1,325,957	1,803,191	789	3497	17,292	21,578	11.0/*11.0*	12.3/*12.3*	12.2/*12.2*	12.1/*12.1*	16.3/*16.3*	15.7/*15.7*	20.1/*20.1*	19.2/*19.2*
Non-purulent SSTI	72,825	443,730	4,246,010	4,762,565	1426	8698	170,065	180,189	44.2/*0.0*	58.4/*0.0*	56.0/*0.0*	56.0/*0.0*	81.5/*0.0*	83.7/*0.0*	73.0/*0.0*	73.6/*0.0*
Otitis externa	-	-	2,121,058	2,121,058	-	-	6707	6707	-	-	9.9/*8.9*	9.9/*8.9*	-	-	42.9/*41.9*	42.9/*41.9*
Pneumonia	99,460	215,402	2,210,882	2,525,744	7219	12,909	77,410	97,538	57.2/*0.0*	64.9/*0.0*	38.5/*0.0*	41.5/*0.0*	84.7/*0.0*	92.3/*0.0*	66.4/*0.0*	71.2/*0.0*
Prostatitis	-	-	379,608	379,608	-	-	2312	2312	-	-	34.0/*0.0*	34.0/*0.0*	-	-	74.1/*0.0*	74.1/*0.0*
Purulent SSTI	106,928	571,744	6,285,648	6,964,320	1145	5257	45,136	51,538	33.0/*0.0*	39.4/*0.0*	18.8/*0.0*	20.7/*0.0*	64.3/*14.3*	63.6/*13.6*	65.5/*35.5*	65.3/*32.8*
Pyelonephritis	15,826	31,366	348,099	395,291	157	1742	29,232	31,131	43.8/*0.0*	54.1/*0.0*	50.6/*0.0*	50.6/*0.0*	89.8/*0.0*	87.9/*0.0*	77.9/*0.0*	78.5/*0.0*
Reproductive tract infections	6443	126,475	2,369,848	2,502,766	-	169	2585	2754	6.5/*0.0*	16.1/*0.0*	14.6/*0.0*	14.7/*0.0*	-	93.9/*0.0*	93.6/*0.0*	93.5/*0.0*
Urinary tract infections	98,858	858,535	9,724,013	10,681,406	4528	11,506	118,149	134,183	42.8/*0.0*	60.4/*0.0*	63.1/*0.0*	62.7/*0.0*	80.8/*0.0*	84.6/*0.0*	83.9/*0.0*	83.9/*0.0*

^1^ Expected rates of prescribing (Wu et al., 2020) are: prescribing rate % minus unnecessary rate %; dashed lines [-] included in cells where not applicable.

## Data Availability

Restrictions apply to the availability of these data. Data were obtained from Population Data BC.
